# Ultrasound and enzyme extraction of *Pleioblastus amarus* shoot polysaccharide: A comparative study on chemical characterization, antioxidant, and anti-aging activities

**DOI:** 10.1016/j.ultsonch.2026.107880

**Published:** 2026-05-04

**Authors:** Ruiqing Han, Yi Gu, Yiming Xu, Siyu Wu, Wenshan Li, Shibo Nan, Yuanfeng Zou

**Affiliations:** Natural Medicine Research Center, College of Veterinary Medicine, Sichuan Agricultural University, Huimin Road 211, Chengdu 611130, Sichuan Province, PR China

**Keywords:** *Pleioblastus amarus*, Polysaccharide, Extraction method, Ultrasound-enzyme extraction, Antioxidant activity, Anti-aging activity

## Abstract

*Pleioblastus amarus* shoot is a critical dietary staple for the giant panda and a traditional medicinal resource for preventing senility, with polysaccharides being one of its primary active ingredients. In this study, we extracted polysaccharides using five distinct extraction techniques, including hot water (HWA-BSP), ultrasound (UA-BSP), enzyme (EA-BSP), and two sequential combinations of ultrasound-enzyme (UEA-BSP) and enzyme-ultrasound (EUA-BSP), and systematically investigated the effects of different extraction method on yield, structural characteristics, and biological activities of the five polysaccharides. The extraction method with superior anti-oxidant ability was identified and its anti-aging efficacy was subsequently evaluated through *in vivo* experiments. Our results demonstrated that while all five polysaccharides shared similar monosaccharide compositions and triple-helical structures, the UEA-BSP method achieved the highest yield (4.73%) and the lowest molecular weight. It also showed the greatest scavenging abilities of DPPH, ABTS, and hydroxyl radical, and superior protection effect on IPEC-J2 cells against H_2_O_2_-induced oxidative injury. Therefore, UEA-BSP was selected to confirm its anti-aging ability in D-galactose induced aging mice. UEA-BSP restored: 1) cognitive function (Morris water maze), 2) hippocampal morphology (H&E staining), and 3) antioxidant enzyme activities (CAT, SOD, GSH-Px) along with MDA level, confirming its potent anti-aging bioactivity. Overall, this study demonstrates that sequential ultrasound-enzyme extraction significantly enhances polysaccharide yield and antioxidant activity, providing valuable insights and a foundational data reference for further investigations into the anti-aging potential of *P. amarus* polysaccharides in functional food and nutritional applications.

## Highlights

• Comparing five extraction methods for Pleioblastus amarus polysaccharides.
• Ultrasound-enzyme extraction exhibited the highest yield and antioxidant activity.
• It restored learning and memory abilities and anti-oxidant enzyme activities in aging mice.

## Graphical abstract

**Figure:**
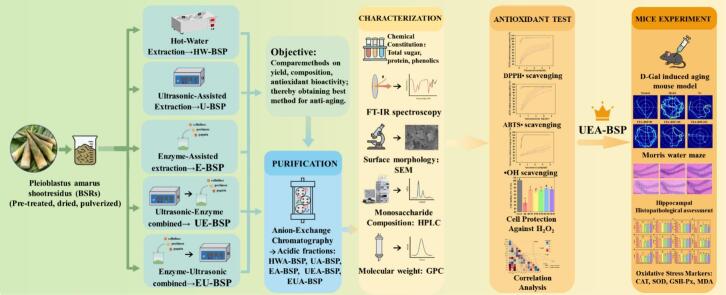

## 1 Introduction

Aging is a complex, multifactorial biological process characterized by a progressive decline in physiological function and a systemic disruption of homeostatic balance [1]. A central driver of this process, beyond chronological age, is chronic low-grade oxidative stress [2]. Aging-associated oxidative stress primarily stems from mitochondrial dysfunction, resulting in the excessive accumulation of reactive oxygen species (ROS). These ROS induce lipid peroxidation, disrupt redox homeostasis, and promote cellular senescence markers, exacerbating age-related tissue damage [3].

*Pleioblastus amarus*, a bamboo species widely distributed in southern China, serves as a preferred dietary staple for the giant panda. It is also extensively consumed by local residents due to its crisp texture and high nutritional value. In addition, this bamboo species is used in traditional medicine for the prevention of senility [4]. Previous studies showed that *P. amarus* shoots are rich in polysaccharides, phenolics and alkaloids [5], [6], and a recent study demostrated that its shoot shells polysaccharides exhibited a superior anti-melanoma activity [7]. Meanwhile, polysaccharides from the shoot of bamboo species like *Phyllostachys edulis* and *Chimonobambusa quadrangularis* were demonstrated to have antioxidant effects [8], [9]. However, the antioxidant and anti-aging properties of *P. amarus* shoot, along with their action mechanisms and composition-activity relationships, have yet to be fully elucidated.

Crucially, extraction methods, including hot water extraction [10], ultrasound extraction [11], enzyme extraction [12], acid/ alkali extraction [13], microwave-assisted extraction [14], deep eutectic solvents extraction [15], [16], can significantly alter the polysaccharide properties. It can shape molecular weight and monosaccharide profile, thus modulating their therapeutic potential [17]. Traditional hot water extraction is still the most commonly used technique; however, it suffers from low yield, high energy consumption, and prolonged processing time [18]. In contrast, ultrasound extraction utilizes cavitation effects to disrupt cell walls, enhance solvent penetration, and promote polysaccharide release. This is achieved through the generation of microbubbles that collapse violently, producing localized extreme conditions, including temperatures up to ∼ 5000 K and pressures reaching ∼ 1000 bar. Consequently, polysaccharides extracted by ultrasound extraction were characterized by higher extraction efficiency, shorter processing times, and improved bioactivity [19]. Enzyme extraction, typically employing cellulase, papain and pectinase, enhances yield and preserves polysaccharide structural integrity [20]. Cellulase hydrolyzes the cellulose framework, compromising cell wall integrity, papain disrupts polysaccharide-protein complexes and eliminates protein impurities, and pectinase degrades pectin, dismantling intercellular connections and improving mass transfer permeability [21], [22], [23]. Collectively, these enzymes synergistically facilitate polysaccharide release, thereby enhancing extraction yield.

Ultrasound and enzyme combined extraction, as a novel extraction method, can synergize ultrasonic cavitation with enzymatic hydrolysis to disrupt cell walls effectively, enhancing polysaccharide yield, reducing molecular weight, and improving bioactivity. Song et al. demonstrated its superiority over traditional hot-water extraction for litchi seed polysaccharides, achieving higher yield and enhancing biological activity [23]. However, though low-power ultrasound can activate enzyme activity, prolonged and high-intensity ultrasonic treatment generates substantial free radicals and exerts strong shear forces, which disrupt the structural integrity of enzyme and diminish its enzymatic activity [24], [25]. Szabo and Csiszar (2013) observed 25% loss in the cellulase activity at ultrasound intensity of 43.4 W [26]. Therefore, although higher ultrasonic power can achieve more thorough cell wall disruption, the reduction in enzyme activity limits the applicable power level [21].

Consequently, the sequential integration of ultrasound and enzyme extraction can fully leverage their extraction potential [27]. Prior studies have implemented diverse processing sequences: for instance, *Gastrodia elata* and *Saposhnikovia divaricate* polysaccharides were extracted using ultrasound followed by enzyme treatment [28], [29], whereas enzymatic hydrolysis was performed before ultrasound for the extraction of polysaccharides from *sea buckthorn* and the *Taraxacum genus* [30], [31]. Meanwhile, the influence of enzyme and ultrasound treatment sequence on the gel strength of oat proteins and enzyme activity of pectinase were investigated [32], [33]. However, a systematic understanding of the effects and underlying mechanisms of sequential processing order on the yield and bioactivity of polysaccharides is still lacking.

In this study, *Pleioblastus amarus* shoots were collected from Yibin City and the objectives of this study are to: 1) compare the yield, chemical composition, molecular weight, monosaccharide composition, and triple-helix structure of polysaccharides extracted by hot water extraction, ultrasound extraction, enzyme extraction, ultrasound-enzyme sequential extraction, and enzyme-ultrasound sequential extraction. 2) compare the antioxidant activities of five polysaccharides by radicals scavenging experiments and cell experiments. 3) evaluate the anti-aging effect using a D-galactose induced aging mouse model of the polysaccharide extracted by the method with the best antioxidant activity, with validation through the Morris water maze test, H&E staining of hippocampus, and antioxidant enzyme assay. This research will deepen insights on the different extraction methods, especially ultrasound and enzyme combined extraction, and provide useful data about the anti-aging effectiveness of *P. amarus* shoot polysaccharides for the further study.

## 2 Materials and methods

### 2.1 Pretreatment of *P. Amarus* shoot

The shoot residues of *P. amarus* were obtained from Yibin City, Sichuan Province, China. It was washed, dried at 60 °C, pulverized to a fine powder. Then, it was mixed with 96% ethanol at 70 °C for 2 h in a ratio of 1:20 (w/v). After filtration, the pretreated residue was dried and sealed.

### 2.2 Extraction methods of bamboo shoot polysaccharides (BSPs)

The BSPs were extracted by the following five methods.

**Hot water extraction method.** The pretreated powder was combined with distilled water (1:20 g/ml) and extracted in a boiling water bath for 90 min [3].

**Ultrasonic extraction method.** The pretreated powder was combined with distilled water (1:20, g/mL), and extraction polysaccharides using an ultrasonic processor (40 kHz; KH-300DE, Kunshan Hechuang, China). The ultrasonic power was maintained at 300 W, with the extraction temperature and duration fixed at 50 °C and 90 min, respectively [25], [34].

**Enzyme extraction method.** The pretreated powder was extracted with distilled water 1:20 (g/mL). Subsequently, a 1% composite enzyme mixture, consisting of cellulase (50 U/mg), papain (800 U/mg), and pectinase (50 U/mg) (1:1:1, w/w/w), was introduced and the pH was adjusted to 5.0. All enzymes were purchased from Shanghai Yuanye Bio-Technology Co., Ltd. (Shanghai, China). Enzyme extraction proceeded at 50 °C for 90 min, followed by a rapid temperature elevation to 100 °C for 15 min to terminate enzymatic activity [25], [34].

Enzyme-Ultrasonic method

The enzyme extraction was conducted for 60 min, and the mixture was heated to 100 °C for 15 min to deactivate the enzyme. Then, ultrasound extraction was conducted for another 30 min under conditions same with Ultrasonic extraction method [25], [34].

**Ultrasonic-Enzyme method.** The ultrasonic extraction was conducted for 30 min under conditions same with Ultrasonic extraction method, and then, the enzyme extraction was conducted for another 60 min, followed by a rapid temperature elevation to 100 °C for 15 min to terminate enzymatic activity [25], [34].

The five extracts were centrifuged, concentrated and mixed with four volumes of absolute ethanol, then stay at 4 °C for 12 h. Following a subsequent centrifugation (5,500 rpm, 15 min), the supernatants were discarded; the precipitates were then recovered and redissolved in water. The aqueous solution was deproteinized via the Sevag method, dialyzed against deionized water, and finally lyophilized. The extracted crude polysaccharides were named HW-BSP, U-BSP, E-BSP, UE-BSP EU-BSP, respectively. Yields were calculated according to the following formula:

(1) $$Polysaccharide\;yield\;\%=\frac{weight\;of\;dried\;polysaccharide\;\left(g\right)}{weight\;of\;pretreated\;powder\;\left(g\right)}$$

### 2.3 Purification of polysaccharides

The crude extracts were fractionated via anion-exchange chromatography using a column packed with DEAE-Sepharose Fast Flow. low yield neutral fractions were eluted and homogeneous acidic fractions were pooled. Elution curves were showed in Fig. S2. Five kinds of acidic polysaccharides were named as HWA-BSP, UA-BSP, EA-BSP, UEA-BSP, EUA-BSP, respectively.

### 2.4 Component and characterization of BSPs

#### 2.4.1 Total sugar, protein and phenolics analysis

Sugar content was assayed via the phenol–sulfuric acid method. Protein and phenolics content of BSPs were determined by Coomassie Brilliant Blue (Bradford) method, Folin-Ciocalteu method, respectively [35].

#### 2.4.2 FT-IR and UV–Vis spectroscopy

3 Mg of BSPs were combined with a certain amount of dry KBr and mixture and obtain the sample spectrum using a Fourier transform infrared (FT-IR) spectrometer. The spectra were recorded within 500–4000 cm^−1^. Additionally, each spectrum (baseline calibration and smoothing) was preprocessed and was plotted. Each acidic polysaccharide sample was dissolved in water and scanned with UV–vis at the wavelength from 200 to 400 nm

#### 2.4.3 Congo red test

The triple-helical conformation of BSPs was characterized [17]. Briefly, BSPs were dissolved in varying concentrations of sodium hydroxide (0.0, 0.1, 0.2, 0.3, 0.4, and 0.5 mol/L) to achieve a concentration of 1.6 mg/L. Polysaccharide solutions (1 mL) were combined with 1 mL of Congo red reagent (80 µmol/L) and allowed to react for 5 min following thorough homogenization. The absorbance maxima were determined by scanning across the 400–650 nm range, with a polysaccharide-free Congo red solution serving as the blank control.

#### 2.4.4 Molecular weight analysis

The molecular weight characteristics of the BSPs were determined via high-performance gel permeation chromatography (HPGPC). Analysis was performed on a Waters Ultra hydrogel Linear column, with calibration achieved using a series of pullulan standards of known molecular weights. The mobile phase was 0.1 M NaNO_3_ aqueous solution, the flow rate was set to 1.0 mL/min, the column temperature was maintained at 35 °C, and the injection volume was 20.00 μL [36].

#### 2.4.5 Monosaccharide composition

BSPs were hydrolyzed with trifluoroacetic acid and derivatized with 1-phenyl-3-methyl-5-pyrazolone. A Waters 2695 HPLC system with a Waters 2996 UV detector was used to analyze with a mobile phase of phosphate buffer and acetonitrile (83:17, v/v), the flow rate was set to 1.0 mL/min, the column temperature was maintained at 30 °C, the detection wavelength was 254 nm, and the injection volume was 20 μL [37].

#### 2.4.6 Surface morphology analysis

The morphological features of the BSPs were visualized using scanning electron microscopy. Sample powders were sputter-coated with a thin layer of platinum to ensure electrical conductivity prior to imaging. Micrographs were acquired across a range of magnifications to characterize the surface topography.

### 2.5 Antioxidant activity

#### 2.5.1 DPPH free radicals scavenging ability

100 μL of a freshly prepared ethanol solution of DPPH (0.2 mmol/L) was combined with equal volumes of BSP solutions at varying concentrations. The absorbance was recorded at 517 nm. The scavenging efficiency was quantified according to the following equation:

(2) $$\text{DPPH free radicals scavenging rate (\%) = 1-}\;\left[\frac{\mathrm{Asample}\;-\;\mathrm{Ablank}}{\mathrm{Acontrol}}\right]$$

Where Asample represents the absorbance of the mixture containing the BSP solution and an equal volume of the DPPH reagent; Ablank denotes the absorbance of the BSP solution mixed with an equal volume of ethanol (to account for intrinsic sample color); and Acontrol corresponds to the absorbance of a mixture comprising deionized water and the DPPH reagent.

#### 2.5.2 ABTS free radicals scavenging ability

3 mmol/L ABTS and 2.45 mmol/L potassium persulfate were mixed with equal volume. This radical solution (170 μL) was subsequently reacted with 30 μL of polysaccharide solution. The absorbance of each mixture was recorded at 734 nm. The scavenging efficiency was quantified using the formula specified in section 2.5.1

#### 2.5.3 Hydroxyl free radicals scavenging activity

To assess hydroxyl radical scavenging, five polysaccharide samples at various concentrations were combined with equal volumes of 6 mM FeSO_4_, 6 mM salicylic acid (in ethanol), and 6 mM H_2_O_2_. The absorbance was measured at 510 nm [38].

#### 2.5.4 Antioxidant assays in IPEC-J2 cells stimulated by H_2_O_2_

Intestinal porcine epithelial cells (IPEC-J2) were seeded in 96-well plates at a concentration of 5 × 10^3^ cells per well and maintained under conditions of 37 °C, 5% CO_2_. Cells were co-incubated with 0.2 mmol/L H_2_O_2_ for 24 h, followed by treatment with BSPs (final concentration of 200 μg/mL) for an additional 24 h. A CCK-8 assay was subsequently used to quantify cell viability. The absorbance was measured at 450 nm, and viability was calculated using the following formula:

(3) $$\text{Cell viability rate (\%) = }\frac{\mathrm{Atest}-\;\mathrm{Ablank}}{\mathrm{Acontrol}-\mathrm{Ablank}}$$

Where Ablank denotes the absorbance of the wells containing culture medium and CCK-8 without cells, Acontrol represents the absorbance of the wells containing cells and CCK-8 solution, and Atest refers to the absorbance of the wells treated with BSPs.

### 2.6 Animals and treatment

4 Male C57BL/6 mice (19 ± 1 g, 8 weeks old) were randomly assigned to six groups (n = 10), including: Control group, D-galactose group, positive group (100 mg/kg Vitamin C), and three UEA-BSP treatment groups (50, 100, and 200 mg/kg/d)

To establish the accelerated aging model, the mice of model, Vitamin C group and polysaccharide groups were injected D-galactose (400 mg/kg/d) for 6 weeks, and equal volume of physiological saline for control group. Concurrently, the Vitamin C and UEA-BSP groups were administered via oral gavage, whereas the same volume of saline was given to control and model group. Body weights were recorded weekly throughout the 42-day intervention.

#### 2.6.1 Morris water maze test

Spatial learning and memory abilities were evaluated by Morris water maze (MWM) task. The experiment was conducted in a 35 cm depth pool, with a 1 cm below the surface platform positioned in the northeast quadrant. During five-day navigation trial, mice underwent four daily sessions, released from varying quadrants to locate the hidden platform. Mice failing to reach the target in 90 s were manually guided to the platform and stay there for 30 s; in such instances, an escape latency of 90 s was recorded. Conversely, mice that successfully located the platform within the allotted time were permitted to remain for 10 s. On day 5, the platform was removed for the spatial probe trial. Mice were introduced from the southwest quadrant and monitored for 90 s, during which swimming trajectories and the frequency of crossings into the former platform area and the time spent in target quadrant were quantified.

#### 2.6.2 Histopathological analysis

Hippocampus tissues were fixed in 10% formaldehyde, dehydrated, embedded, sliced, and stained by H&E.

#### 2.6.3 Biochemical analyses

All mice were sacrificed for biochemical analysis. Blood, brain and liver samples were collected and commercial kits were used to quantified the activities of superoxide dismutase (SOD), catalase (CAT), and glutathione peroxidase (GSH-Px) along with the levels of malondialdehyde (MDA).

### 2.7 Statistical analysis

For cell assays and *in vivo* experiments, a sample size of n = 10 per group was employed as biological replicates, with the exception of antioxidant enzyme and AChE activity assays, which utilized n = 6 randomly chosen samples per group from each group. Other *in vitro* assays were performed in triplicate as technical replicates. All data are presented as mean ± SD. Statistical analysis was conducted by one-way ANOVA with post hoc multiple comparisons using SPSS 22.0. Graphs were plotted using Origin 2021 and R 3.2.0. *P <* 0.05 was considered statistically significant (*P < 0.05, **P < 0.01, ***P < 0.001).

## 3 Results and discussion

### 3.1 Impact of extraction methods on polysaccharide yield

**Fig. 1:**
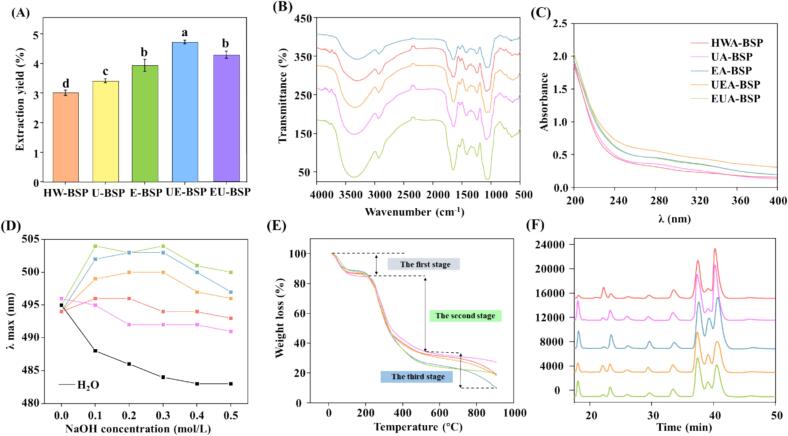
Polysaccharide yield, characteristics, and monosaccharide composition of five bamboo shoot polysaccharides (BSPs). (A) Effect of different extraction methods on the extraction rate of BSPs. The characteristics of BSPs, including Fourier transform infrared (FT-IR) spectra (B), UV-scanning spectra (C), Congo red experiment (D) Thermogravimetric curve (E), and monosaccharide composition by HPLC chromatograms (F). The BSPs were named according to different extraction methods: hot water extraction (HWA-BSP), ultrasound extraction (UA-BSP), enzyme extraction (EA-BSP), ultrasound-enzyme extraction (UEA-BSP), enzyme-ultrasound extraction (EUA-BSP). Samples labeled with the same letter are not significantly different at 0.05 level.

This study investigated the extraction yields of polysaccharides of five extraction methods, and results showed 3.01% of conventional hot water extraction, 3.94% of enzyme extraction, 3.41% of ultrasound extraction, 4.73% of ultrasound-enzyme extraction, and 4.28% enzyme-ultrasound extraction (Fig. 1A). The sequential utilization of enzyme and ultrasound was beneficial for the yield of polysaccharides from *P. amarus* shoot polysaccharides. Combined effects between ultrasonic cavitation and enzymatic hydrolysis enhance polysaccharide release by efficiently breaking cell walls and solubilizing polymer [32].

The comparison of polysaccharide extraction yield of enzymatic extraction and ultrasound extraction have been widely reported [17], [20], [39], but until now, only few studies explored the order of enzyme and ultrasound when they were sequentially used [25].

We found the ultrasound-enzyme extraction achieved higher polysaccharide yield compared to the enzyme-ultrasound approach, this superior performance may be attributed to the ultrasonic pretreatment effectively disrupting the rigid cellular structure of bamboo shoots through cavitation effects, thereby creating micro-fissures that facilitate subsequent enzymatic penetration and hydrolysis [33], [40], [41]. It is interesting that Hu et al. (2025) found enzyme-ultrasound extraction exhibited better yield of pumpkin seeds compared to ultrasound-enzyme extraction, which was different with this study [25]. The possible reason was the differences in plant tissue structure. Bamboo shoots possess a dense, cellulose-rich matrix that benefits from initial ultrasound treatment, while pumpkin seeds may contain more accessible storage polysaccharides that respond better to direct enzymatic attack. However, it should be noted that Single-Factor experiments or Response Surface Methodology (RSM) are typically required to further optimize the extraction parameters for maximizing polysaccharide yields. Besides, it should be noted that additional control experiments, such as ultrasound-only pretreatment followed by non-enzymatic incubation or enzymatic hydrolysis excluding thermal inactivation effects, are warranted in future studies to independently evaluate the individual contributions of each treatment.

### 3.2 Chemical characteristics

As shown in Table 1 and Fig. S2, the elution curves of all five BSPs exhibited a single, symmetrical peak, and their total carbohydrate contents ranged from 70.52% to 74.52%. These results collectively demonstrated the high degree of homogeneity and purity of the obtained polysaccharides. As showed in Table 1, the minor protein content (1.52%–2.43%) was detected by the Bradford assay. However, it was not prominent in the UV–Vis spectra (Fig. 1C), which could be attributed to the sensitivity threshold of UV detection at 280 nm for low protein concentrations [42]. Phenolic contents remained minimal across all samples (<0.7%). Considering non-covalently bound polyphenols were removed during the alcohol precipitation and dialysis steps, the small amount of remaining polyphenols is likely to be covalently bound [43].

**Table 1:** The chemical characteristics and molecular weight of BSPs.

	HWA-BSP	UA-BSP	EA-BSP	UEA-BSP	EUA-BSP
Chemical composition (%)					
Sugar (%)	70.52 ± 0.61	71.48 ± 0.35	72.23 ± 0.66	74.16 ± 1.16	74.52 ± 0.73
Protein (%)	1.52 ± 0.09	1.63 ± 0.17	2.43 ± 0.11	2.02 ± 0.09	2.22 ± 0.04
Phenolics (%)	0.30 ± 0.01	0.51 ± 0.01	0.48 ± 0.01	0.66 ± 0.03	0.58 ± 0.03
Monosaccharide composition (mol/%)					
Man	1.80	7.21	5.27	3.83	6.82
Rib	5.47	1.72	1.26	2.82	1.74
Rha	1.36	4.02	5.30	3.43	4.52
GlcA	6.04	6.62	4.77	6.18	6.22
GalA	8.29	7.52	7.84	9.15	7.94
Glc	6.99	4.35	1.92	3.60	4.03
Gal	20.07	20.87	16.39	22.20	20.27
Xyl	11.39	11.38	24.51	17.41	18.40
Ara	38.57	36.31	32.74	31.38	30.06
Molecular weight (Da)					
MW	82 838	33 598	28 314	23 468	25 430
MN	6896	2753	2773	2135	2518
MW/Mn	12.01	12.20	10.21	10.99	10.10

Man: mannose; Rib: ribose; Rha: rhamnose; GlcA: glucuronic acid; GalA: galacturonic acid; Glc: glucose; Gal: galactose; Xyl: xylose; Ara: arabinose.

At FT-IR spectrums (Fig. 1B), a similar spectroscopy was observed across different polysaccharides, showing peaks around 3400 cm^−1^ (stretching vibration of O-H), 2933 cm^−1^ (stretching vibration of C-H), 1651 cm^−1^ (stretching vibration of − C=O), and 1423 cm^−1^ (band vibration of C-H) [38]. The absorption peaks at 1040 cm^−1^ and 1075 cm^−1^ are characteristic of pyranose ring vibrations in polysaccharides, corresponding to C-O-C stretching and skeletal vibrations [18]. UV spectra of BSPs (Fig. 1C) showed no significant absorption at 260 nm, suggesting that nucleic acid contaminants were undetectable at this method [44].

The Congo red assay was employed to investigate the triple-helical structure of polysaccharides. As shown in Fig. 1D, all five polysaccharides samples extracted by different methods exhibited distinct red shift relative to the blank Congo red curve across the tested NaOH concentration range (0–0.5  mol/L), confirming that all polysaccharides possess a triple-helical structure. This suggests both enzyme and ultrasound extractions can preserve the triple-helical conformation, aligning with the previous study [45]. It should be noted that while the Congo red assay provided preliminary evidence for a triple-helix structure, further validation using complementary techniques such as Circular Dichroism (CD) spectroscopy or X-ray diffraction (XRD) would be beneficial in future studies to provide more direct structural confirmation [46], [47], [48], [49], [50].

However, as FT-IR, UV spectra, and Congo red only provide preliminary information on functional groups, purity, and conformational features, the precise glycosidic linkages and configurations of BSPs need to be further investigated via methylation and NMR analysis in future studies.

### 3.3 Thermal stability analysis

Thermal stability of polysaccharide is a fundamental physicochemical property that dictates their suitability for various practical and industrial applications [3]. Thermogravimetric profiles (Fig. 1E) exhibited that all BSPs exhibited a similar and progressive mass reduction between 0 °C and 900 °C, characterizing a three-stage process. The first stage involved a marginal weight decrement, primarily associated with the liberation of both physically adsorbed and chemically bound water [51]. The subsequent primary degradation stage was marked by accelerated weight loss, predominantly driven by the cleavage of glycosidic linkages and the disintegration of side-chain residues [52]. The final stage corresponded to the further thermal breakdown of carbonaceous residues and depolymerization products [53].

### 3.4 Monosaccharide composition

The monosaccharide compositions (Fig. 1F and table 1), showed that the extraction techniques did not alter the qualitative monosaccharide types, with all samples consisted of the same nine monosaccharides: mannose (Man), ribose (Rib), rhamnose (Rha), glucuronic acid (GlcA), galacturonic acid (GalA), glucose (Glc), galactose (Gal), xylose (Xyl), and arabinose (Ara). However, the molar proportions of these monosaccharides were significantly influenced by the extraction methods. Specifically, BSPs were primarily composed of arabinose (30.06%–38.57%), galactose (16.39%–22.20%), and xylose (11.38%–24.51%). In contrast to the *P. amarus* shoot polysaccharide reported by Ren et al. [54], our results showed a higher content of arabinose, whereas fucose was not detected.

### 3.5 Microscopic characteristics

**Fig. 2:**
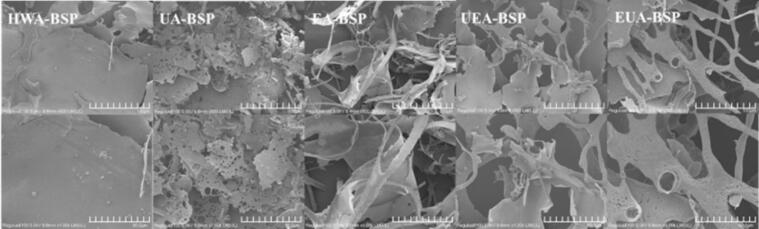
Scanning electron microphotograph (magnification 500 × and 1000 × ) of BSPs extracted by different methods.

As illustrated in Fig. 2, HWA-BSP displayed a folded, sheet-like morphology, because hot water extraction likely preserved the initial structure [39], [55]. In contrast, a porous, paper-like shape was observed in ultrasonic treatment. Ultrasonic relies on the cavitation effect, characterized by the nucleation and subsequent implosion of microbubbles, which generates intense micro-scale shockwaves and localized high-pressure zone. This process facilitates the depolymerization of polysaccharides and induces the formation of numerous voids[19]. EA-BSP displayed a dual morphological configuration, comprising a folded, paper-like morphology and a slender structure. It exhibited a more fragmented structure, likely due to enzymatic hydrolysis of glycosidic linkages, producing shorter polymer chains with reduced molecular weight [3]. UEA-BSP and EUA-BSP exhibit a hybrid structural feature from two extraction techniques. These samples display porous regions caused by ultrasonic cavitation, along with the folded, sheet-like, and striped patterns caused by enzymatic hydrolysis.

### 3.6 Molecular weight

HPGPC was employed to determine the molecular weights (*Mw*) of the polysaccharides (Table 1 and Fig. S2), following a descending order from HWA-BSP, UA-BSP, and EA-BSP, to EUA-BSP, with UEA-BSP exhibiting the lowest values. Polysaccharides extracted by hot water exhibited the highest *Mw*, suggesting that polysaccharide molecules are prone to aggregation at elevated temperatures [56]. In contrast, ultrasound treatment can reduce *Mw*, which is attributed to two primary mechanisms: 1) physical destruction by microbubbles and 2) oxidative degradation on polysaccharide chains by hydroxyl radicals (•OH). Furthermore, enzyme treatment effectively reduces *Mw* by hydrolyzing glycosidic bonds [19], [39].

Compared with EUA-BSP, UEA-BSP exhibited lower *Mw*, and the potential mechanisms are speculated as follows. Firstly, as shown in Table 1, polysaccharides extracted by enzyme alone exhibited lower *Mw* compared with ultrasound alone, −suggesting that enzymes might possess a superior capacity for molecular weight reduction under these conditions. This observation is consistent with the previous studies [3], [17]. Therefore, when polysaccharides were extracted with ultrasound-enzyme order, ultrasound could degrade cell wall and release polysaccharides, and then enzyme can directly reduce *Mw*. Secondly, as illustrated in Fig. 2, ultrasound creates porous structures with enlarged surface areas, potentially exposing more active sites for subsequent enzymatic hydrolysis [32]. These combined effects likely contribute to the further reduction of molecular weight in UEA-BSP.The polydispersity index (Table 1) serves as a key indicator of the molecular weight distribution breadth in polysaccharides. In this study, all BSP fractions exhibited relatively high values, suggesting a broad molecular weight distribution and indicating that the different extraction methods had minimal impact on the overall polydispersity.

### 3.7 Antioxidant activities

**Fig. 3:**
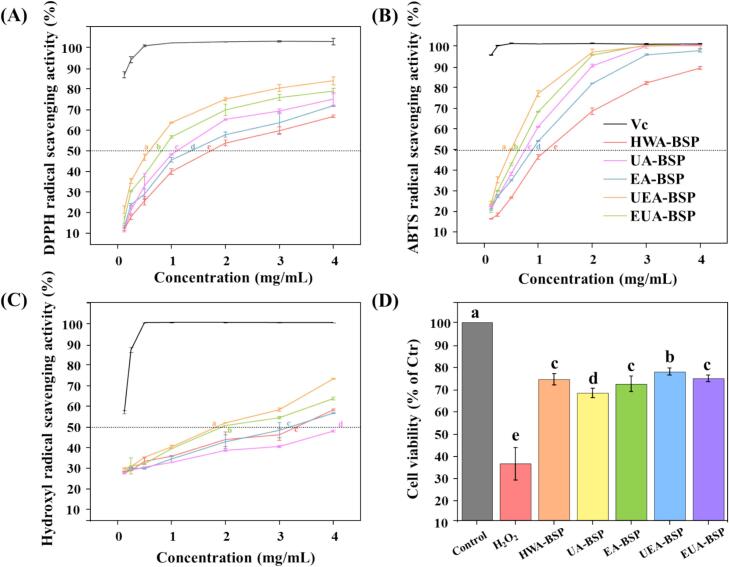
Antioxidant effects of BSPs *in vitro* including DPPH radical (DPPH•) scavenging activity (A), ABTS radical (ABTS•) scavenging activity (B), hydroxyl radical (•OH) scavenging activity (C). (D) The antioxidant defense effect of five BSPs on IPEC-J2 cells (n = 10). Samples labeled with the same letter are not significantly different at 0.05 level.

The antioxidant capacities of the five polysaccharides were evaluated (Fig. 3). All polysaccharides exhibited a significant concentration-dependent increase in their DPPH, ABTS and •OH radicals scavenging capacity. The half-maximal inhibitory concentration (IC_50_) values of DPPH radicals were Vc (0.04 mg/mL), UEA-BSP, (0.68 mg/mL), EUA-BSP (1.06 mg/mL), UA-BSP (1.36 mg/mL), EA-BSP (1.69 mg/mL), and HWA-BSP (2.31 mg/mL) (Fig. S1A). Although the scavenging efficiency of these five polysaccharides was slightly lower than Vc, they demonstrated substantial potential as natural radicals scavengers. A similar trend was observed in ABTS radicals scavenging (Fig. 3B) and the IC_50_ were as follows: Vc (0.03 mg/mL), UEA-BSP (0.67 mg/mL), EUA-BSP (0.89 mg/mL), UA-BSP (1.00 mg/mL), EA-BSP (1.25 mg/mL) and HWA-BSP (1.71 mg/mL) (Fig. S1 high degree B). Furthermore, the hydroxyl radicals scavenging activity was investigated (Fig. S1C), The IC_50_ values were Vc (0.15 mg/mL), UEA-BSP (2.20 mg/mL), EUA-BSP (2.65 mg/mL), EA-BSP (3.44 mg/mL), HWA-BSP (3.49 mg/mL), and UA-BSP (5.13 mg/mL). While all five polysaccharides displayed an upward trend in activity as concentrations increased, their overall efficacy in neutralizing •OH was relatively limited compared to their performance in the DPPH and ABTS radicals. In addition, the triple-helix structure observed in *P. amarus* shoot polysaccharides (Fig. 1D) is hypothesized to potentially enhance its antioxidant bioactivity [39]. However, the specific contribution of this structural feature requires further investigation.

Compared to the *in vitro* radicals scavenging tests, cell experiment can provide more direct evidence on antioxidant activities [35]. Therefore, we employed the IPEC-J2 cell line to investigate these protective effects using H_2_O_2_ oxidative stress model. The IPEC-J2 cell line plays a pivotal role in nutrient absorption and mucosal barrier integrity, making it a robust platform for investigating the attenuation effects of polysaccharides against oxidative injury [35], [57]. As illustrated in Fig. 3D, exposure led to a sharp decline in IPEC-J2 viability, likely stemming from oxidative damage to cellular macromolecules [58]. Conversely, treatment with the five BSPs significantly reversed this trend, demonstrating their robust cytoprotective potential. Notably, a standardized concentration of 200 μg/mL was adopted to compare the relative bioactivities across different extraction groups at this stage, although a detailed dose–response analysis remains highly recommended for future mechanistic explorations.

### 3.8 Relationship between polysaccharide component and antioxidant activities

**Fig. 4:**
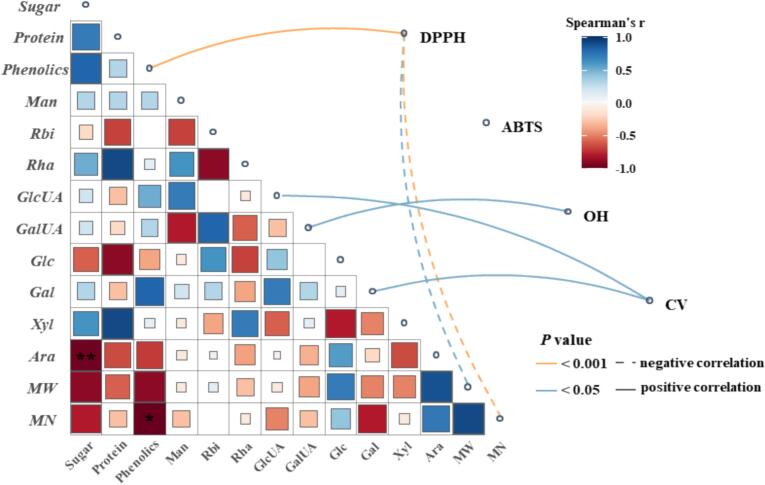
Spearman’s correlation analysis between physicochemical properties and biological activities of polysaccharides. Solid lines represent positive correlations, while dashed lines represent negative correlations. Line colors indicate significance levels: orange (*P* < 0.001) and blue (*P* < 0.05). Abbreviations: Man: mannose; Rbi: ribose; Rha: rhamnose; GlcUA: glucuronic acid; GalUA: galacturonic acid; Glc: glucose; Gal: galactose; Xyl: xylose; Ara: arabinose. CV: cell viability.

To further elucidate the potential relationship between the composition and the antioxidant activities of the polysaccharides, a spearman correlation analysis was performed (Fig. 4). The phenolic content was positively correlated with the DPPH radicals scavenging activity (*P* < 0.001), which was in line with the superior antioxidant bioactivity of polysaccharide-polyphenol conjugates [4]. This suggests that polyphenols may play a critical role in radicals scavenging capacity. Besides, protein may also contribute to anti-oxidant activity, though no significant correlation was observed [59]. Furthermore, the GalA content showed a positive correlation with the hydroxyl radicals scavenging capacity (*P* < 0.05). The enhanced antioxidant activity of polysaccharides with uronic acid is thought to be potentially associated with the selective abstraction of the C-5 hydrogen by hydroxyl radicals, a process that might be facilitated by the steric and stereoelectronic effects of the adjacent electron-withdrawing carboxyl group [59].

Regarding the cellular level of antioxidant defense, the cell viability was found to be positively correlated with the contents of GlcA and Gal (*P* < 0.05), which aligns with previous studies [50]. Overall, polysaccharides with higher contents of GlcA and Gal might possess superior anti-oxidant potential. While these correlations provide valuable insights into the contribution of specific components, it should be noted that they do not inherently prove direct causation, and the complex combined effects between different structural features require further investigation.

Correlation analysis also exhibited the low *Mw* linked to enhanced antioxidant capacity (*P* < 0.05), and similar phenomenon was also reported in *Dendrobium officinale* polysaccharide [3] and comfrey (*Symphytum officinale* L.) root polysaccharide [56]. It could be attributed to the increased exposure of reducing ends in accepting [60]. From *in vitro* view, low molecular weight polysaccharides could be easier to penetrate biological membranes and effectively engages with cellular enzymes and receptors, and may enhance their interaction with biological systems because of superior surface-area-to-volume ratio. As a result, low-molecular-weight polysaccharides may exhibit a stronger capacity to enhance antioxidant enzyme activities [61], [62]. Besides, it could improve solubility enhances bioavailability, leading to higher absorption rates in the digestive tract, and these combined characteristics render low molecular weight polysaccharides possess extraordinary anti-aging bioactivity [61]. Therefore, UEA-BSP exhibited superior radicals scavenging ability and effects of protecting IPEC-J2 against H_2_O_2_ induced oxidative stress, which are likely associated with its high polyphenols content low molecular weight, high content of Gal and GalA.

### 3.9 Antiaging activity in D-gal induced aging mice model

**Fig. 5:**
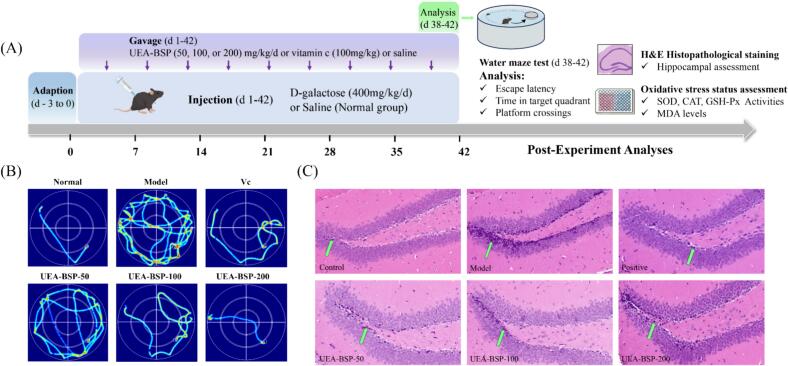
(A) Animal experiment diagram. (B) Typical swimming track visualized of Morris water maze test on day 5. (C) Effects of UEA-BSPs on hippocampal tissue in D-gal induced aging mice visualized by H&E staining. Green arrows indicate nuclear pyknosis.

Considering the superior antioxidant capability and low *Mw* exhibited in UEA-BSP, it was used in the following anti-aging experiment using D-gal induced aging mice model. The experimental workflow is illustrated in Fig. 5A.

#### 3.9.1 Body weight change

**Fig. 6:**
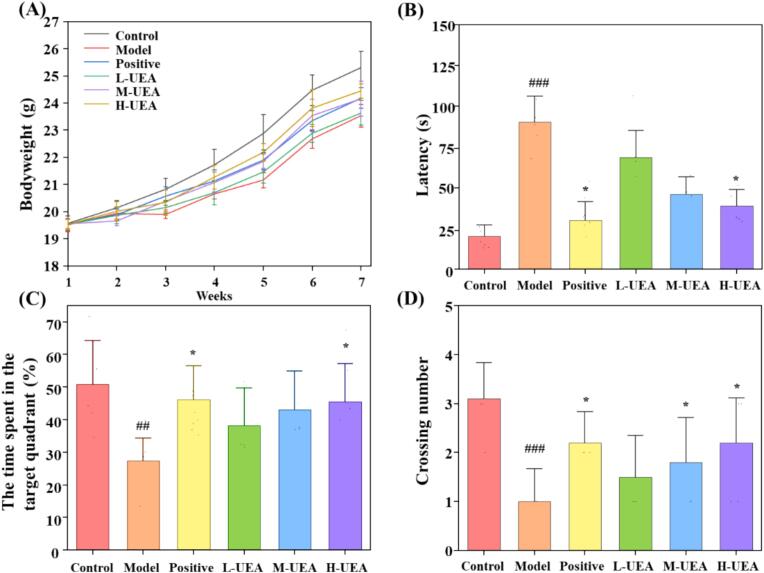
Effect of UEA-BSP on body weight, learning ability in Morris water maze test. (A) The change of body weight with different dose of BSPs. The mitigation of BSPs on learning ability in the D-gal induced aging mice, which quantified by escape latency to find the platform (B), time spent in the quadrant (C), number of target platform crossings (D). Data were expressed as mean ± SD, n = 10. * and ** indicate *P* < 0.05, and *P* < 0.01, respectively, compared with aging model control group induced by D-gal. #, ## and ### indicate *P* < 0.05, *P* < 0.01, and *P* < 0.001, respectively, compared with Model group.

The change in body weight for all experimental mice over the 6-week period is illustrated in Fig. 6A. Although every group showed a gradual increase in weight throughout the period, the D-gal induced model group displayed a lower rate of weight gain compared to the control group. However, treatment with UEA-BSP mitigate this weight inhibition in a dose-dependent manner, and the average body weight in the sixth weeks was 24.44 g and 23.52 g in high-dose group and model group, respectively.

#### 3.9.2 Morris water maze test

The morris water maze (MWM) test serves as a validated behavioral paradigm to evaluate hippocampal-dependent spatial learning and memory in aging models. The cognitive function of D-gal induced aging mice was evaluated using the Morris water maze test, comprising a place navigation trial and a spatial probe trial. In D-gal induced aging mice, prolonged escape latency and reduced platform crossings reflect impaired navigation ability, mirroring cognitive decline observed in mice aging [63], [64]. Aging model group exhibited marked impairments in both spatial learning and memory retention, as evidenced by significantly prolonged escape latency (Fig. 6B, P < 0.001 vs. control group) and reduced time spent in the target quadrant (Fig. 6C, P < 0.001), and typical swimming track was showed in Fig. 5B. In contrast, the positive control (Vc) and the high-dose experimental group showed significant improvements in these behavioral parameters. Notably, the high-dose group exhibited a substantially shortened escape latency (Fig. 6B, P < 0.05 vs. model group) and increased time in the target quadrant (Fig. 6C, P < 0.05 vs. model group), indicating enhanced spatial learning ability. In the spatial probe trial, the number of platform crossings, a key indicator of memory retention, was significantly higher in the high-dose group compared to the model group (Fig. 6D, P < 0.01), with values approaching those of the control group.

#### 3.9.3 Histopathological assessment by H&E staining

Histopathological assessment of the hippocampal appeared to reveal that D-gal treatment induced certain histopathological change, characterized by features such as marked neuronal loss, disordered cellular arrangement, cytoplasmic vacuolization, and pyknotic nucleus. Observationally UEA-BSP treatment seemed to mitigate effectively these pathological alterations in a dose-dependent manner, with an apparent restoration of cellular density and structural integrity. Specifically, mice receiving high-dose UEA-BSP presented relatively compact and orderly granular cell layers with a noticeable reduction in pyknotic neurons, comparable to the morphology observed in the positive groups. These observations suggest that UEA-BSP may exert potent neuroprotective effects by alleviating oxidative stress-mediated neuronal damage and preserving hippocampal microarchitecture in aging mice [63].

#### 3.9.4 Effect of UEA-BSP on antioxidant enzyme activity in D-gal induced aging mice

**Fig. 7:**
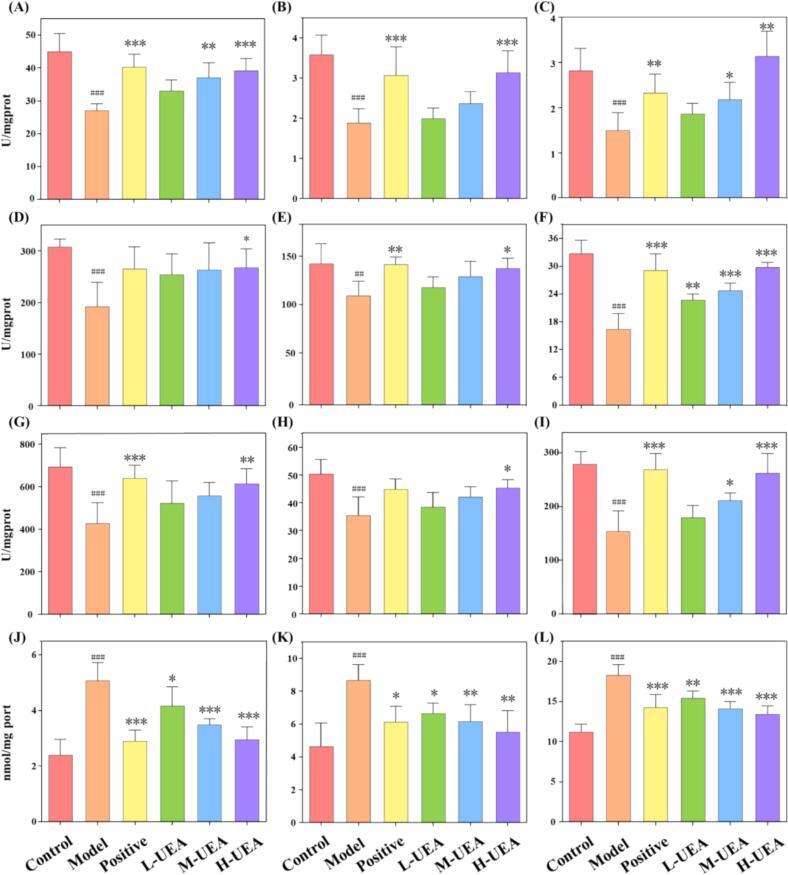
Effect of polysaccharides on activities of CAT (U/mgprot) in liver (A), brain (B), serum (C); SOD (U/mgprot) in liver (D), brain (E), serum (F); GSH-Px (U/mgprot) in liver (G), brain (H), serum (I), and MDA content in liver (J), brain (K), serum (L) in anti-aging experiments. Data were expressed as mean ± SD, n = 6. * and ** indicate *P* < 0.05, and *P* < 0.01, respectively, compared with aging model control group induced by D-gal. #, ## and ### indicate *P* < 0.05, *P* < 0.01, and *P* < 0.001, respectively, compared with Model group.

The D-gal induced aging model is widely recognized for its ability to simulate natural senescence by fostering an environment of excessive free radicals and ROS-mediated oxidative stress [65]. In this study, UEA-BSP intervention effectively reversed these pathological trends in a dose-dependent manner, including the marked restoration of superoxide dismutase (SOD), catalase (CAT), and glutathione peroxidase (GSH-Px) activities in the liver, brain, and serum (Fig. 7A-I). They work synergistically to neutralize ROS. SOD converts superoxide radicals (O_2_•^–^) into hydrogen peroxide (H_2_O_2_), while CAT and GSH-Px further break down H_2_O_2_ into water, thereby preventing the formation of harmful hydroxyl radicals (•OH). It suggests that UEA-BSP enhances the endogenous scavenging of ROS, which corroborated by a significant reduction in the accumulation of malondialdehyde (MDA), a product of polyunsaturated fatty acid oxidation, reflecting the degree of membrane lipid damage caused by ROS [63] (Fig. 7J-L). While this study demonstrates the protective effects of UEA-BSP against aging, the underlying molecular mechanisms remain to be fully elucidated. Future work will be required to focus on investigating the role of UEA-BSP in modulating gut microbiota homeostasis and the activation of the Nrf2 signaling pathway.

#### 3.9.5 Effects of UEA-BSP on the Acetylcholinesterase (AChE) activity

Fig. S1D exhibited that D-gal administration significantly increased brain AChE activity compared to the control group, Notably, the H-UEA group exhibited a statistically significant reduction in enzyme levels compared to the model group.

The cholinergic system is essential for modulating the hippocampal circuits involved in learning and memory [64]. Acetylcholine (ACh), the primary neurotransmitter in this system, plays a critical role in maintaining the efficacy of synaptic transmission. AChE terminates this signaling by catalyzing the hydrolysis of ACh; consequently, elevated AChE activity is frequently associated with cholinergic deficiency and cognitive impairment [66], [67]. In the present study, UEA-BSP treatment significantly inhibited AChE activity in the brain. This reduction in AChE activity potentially mitigates the excessive degradation of ACh, which may contribute to the improved behavioral performance observed in the Morris water maze, such as shortened escape latencies and increased platform crossings. These findings suggest that UEA-BSP may ameliorate cognitive decline by modulating cholinergic enzyme activity, although further studies are required to directly quantify ACh levels and synaptic markers.

## 4 Conclusion

In this study, *P. amarus* shoot polysaccharide was extracted using five methods of ultrasound and/or enzyme (HWA-BSP, UA-BSP, EA-BSP, UEA-BSP, and EUA-BSP), and the obtained polysaccharides were systematically compared for yield, chemical composition and anti-oxidant activity. Results showed that the five obtained polysaccharides possess high content of polysaccharides, low content of protein and polyphenols, and triple-helical structure. Notably, UEA-BSP obtained the highest yield and lowest molecular weight. It also exhibited superior protection effect on IPEC-J2 cells against H_2_O_2_-induced oxidative injury. The anti-aging effects of UEA-BSP was further evaluated using D-gal model aging mice. The Morris water maze test showed that UEA-BSP can protect memory and learning ability, which aligns with its alleviated histopathological change in the hippocampal region. In addition, UEA-BSP significantly enhanced CAT, SOD and GSH-Px activities and reduced MDA levels in serum, liver and brain tissues in aging mice. Overall, this study highlights the order of ultrasound and enzyme during extraction exhibit significant influence on anti-oxidant activity of *P. amarus* shoot polysaccharide, offering a valuable reference for the future application of *P. amarus* polysaccharides as anti-aging functional food ingredients.

Institutional Review Board Statement.

All animal studies and procedures were conducted in accordance with the guidelines and regulations approved by the Ethics Committee of Sichuan Agricultural University (Confirmation number: 20250574).

## CRediT authorship contribution statement

**Ruiqing Han:** Writing – original draft, Software, Methodology, Investigation, Formal analysis, Data curation. **Yi Gu:** Writing – original draft, Methodology. **Yiming Xu:** Methodology, Formal analysis. **Siyu Wu:** Writing – original draft. **Wenshan Li:** Investigation. **Shibo Nan:** Investigation. **Yuanfeng Zou:** Writing – review & editing, Validation, Supervision, Resources, Conceptualization.

## Funding

The authors would like to acknowledge the financial support from the Provincial Undergraduate Training Program on Innovation and Entrepreneurship (No. S202510626029).

## Declaration of competing interest

The authors declare that they have no known competing financial interests or personal relationships that could have appeared to influence the work reported in this paper.
