# Supplementary material for: Ultrasound and enzyme extraction of Pleioblastus amarus shoot polysaccharide: A comparative study on chemical characterization, antioxidant, and anti-aging activities
Source: Ultrason Sonochem. 2026 May 4;129:107880. doi: 10.1016/j.ultsonch.2026.107880 (PMC13156775; doi:10.1016/j.ultsonch.2026.107880)
Supplement: Supplementary Data 1 [file mmc1.docx]

Supplementary data for

Ultrasound and enzyme extraction of *Pleioblastus amarus* shoot polysaccharide: A comparative study on chemical characterization, antioxidant, and anti-aging activities


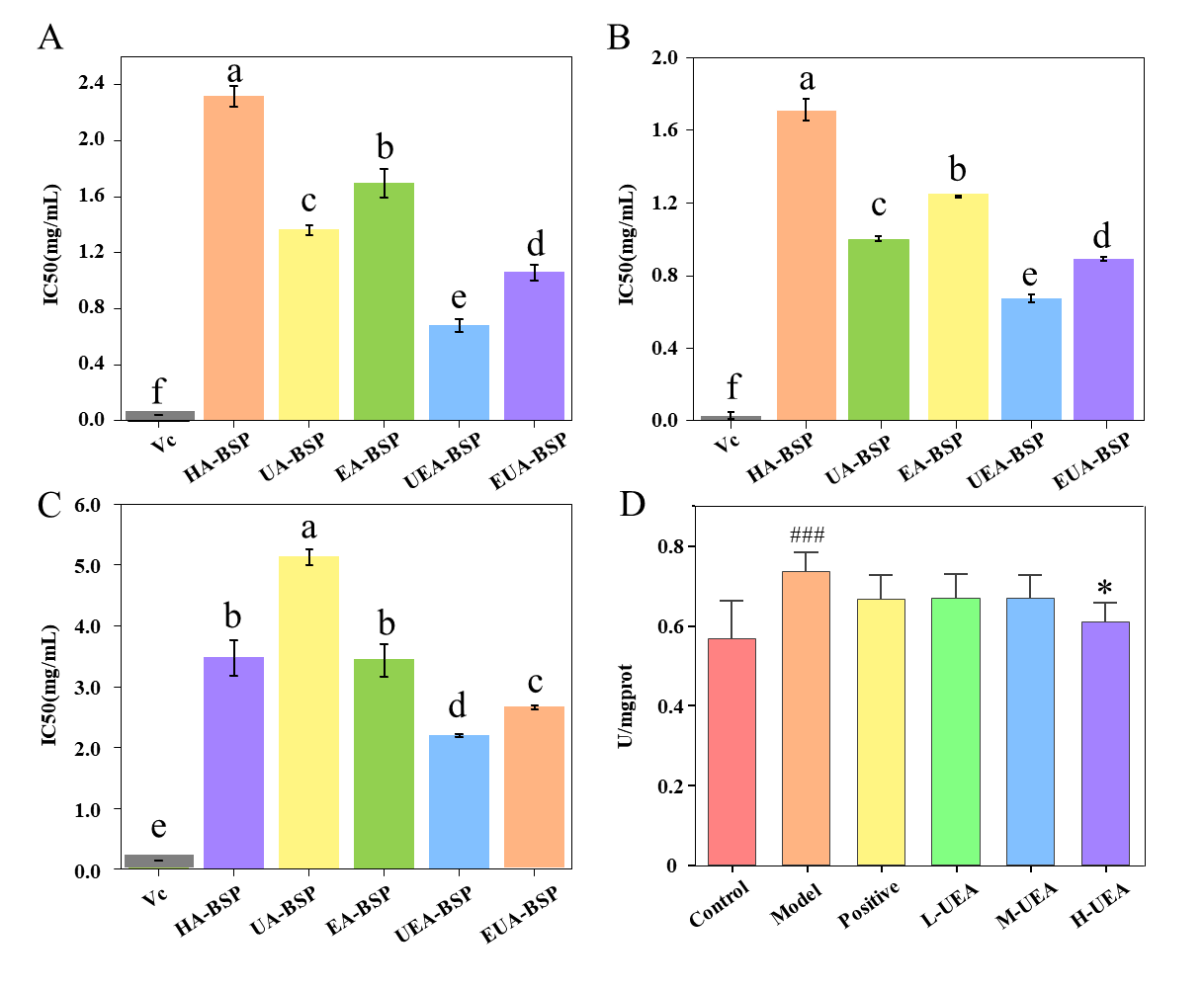


Fig. S1

Scavenging effects on different radicals, DPPH (A), ABTS (B) and OH (C). (D) Effect of polysaccharides on activities of AChE in brain.

Samples labeled with the same letter are not significantly different at 0.05 level. * indicates *P* <0.05 compared with aging model control group induced by D-gal. ### indicates *P* <0.001 compared with Model group.


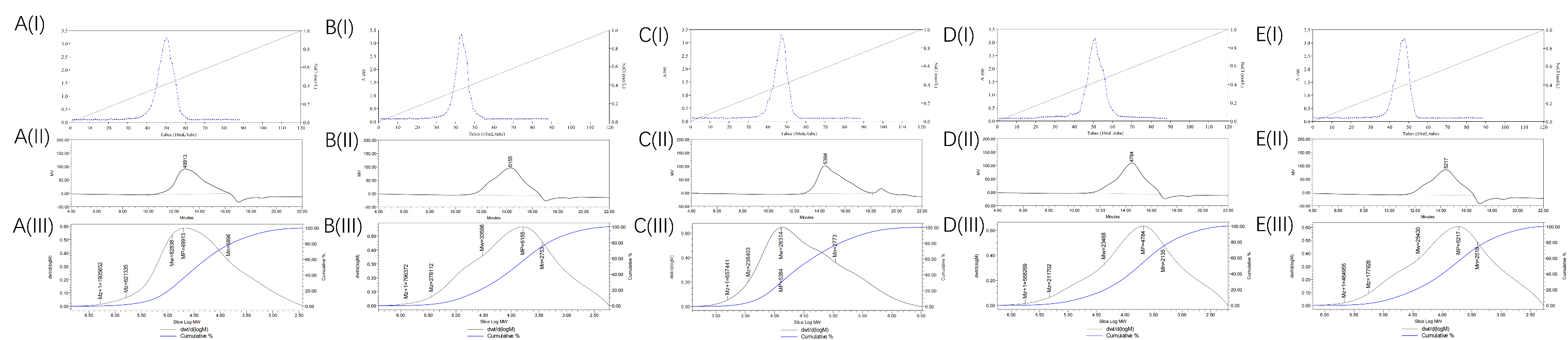


Fig S2

The elution curve (I), Auto-Scaled Chromatogram (II), and Molecular Weight Distribution (III) for HWA-BSP (A), UA-BSP (B), EA-BSP (C), UEA-BSP (D), and EUA-BSP (E).
